# Biomechanical Analysis of Anticipation of Elite and Inexperienced Goalkeepers to Distance Shots in Handball

**DOI:** 10.2478/v10078-012-0062-0

**Published:** 2012-10-23

**Authors:** F. Javier Rojas, Marcos Gutiérrez-Davila, Manuel Ortega, José Campos, Juan Párraga

**Affiliations:** 1 Faculty of Sports Sciences, University of Granada, Spain.; 2 Faculty of Education Sciences, University of Seville, Spain.; 3 Faculty of Physical Activity and Sports Sciences, University of Valencia, Spain.; 4 Faculty of Education Sciences, University of Jaen, Spain.

**Keywords:** biomechanics, anticipation, handball, goalkeeper, throw

## Abstract

The objective of this study was to evaluate the anticipation time and kinematic factors in the movement of goalkeepers’ center of mass when making a long-distance throw in handball. The sample group was composed of 14 goalkeepers and field players. A force platform was used to measure the force of the goalkeepers’ reaction movements, while the throwers’ movements were recorded with high-speed cameras. The expert goalkeepers began to move 193 ± 67 ms before the ball was released, with a 67% success rate of interception. The inexperienced goalkeepers began their movement 209 ± 127 ms with a 24% success rate. The time taken by expert goalkeepers to begin a vertical movement of their CM, relative to the moment of the ball’s release, was less than the time taken by inexperienced goalkeepers (77 ± 70 vs. 141 ± 108 ms respectively). The analysis of the velocity and movement indicates that expert goalkeepers wait longer before moving than do inexperienced goalkeepers.

## Introduction

The ability to intercept objects, which requires highly refined motor abilities and perception skills, is one of the most complex tasks of elite athletes in team sports. Numerous studies confirm that this skill is based on the ability to efficiently use cues from the opponent’s movements in order to predict a precise technical action (Williams et al., 1999; Abernethy and Zawi, 2007; Vignais et al., 2009).

In this sense, one aspect distinguishing better performance of elite players compared with less experienced ones is a superior ability to anticipate the opponent. Previous studies have shown that the ability to anticipate the path of an object in motion is related to the capacity of searching for and identifying directionality indicators (Savelsbergh et al., 2002; McRoberts et al., 2009; Cañal-Bruland et al., 2011) as well as adjusting the temporo-spatial motor response used for interception (Cañal-Bruland and Schmidt, 2009; Nabil and LaRue, 2011; Gutiérrez-Dávila et al., 2011).

Goulet et al. (1989) analyzed the visual reactions of expert and novice tennis players receiving a serve to identify directionality indicators. Expert tennis players focused their eye on the movement of the racket and the opponent’s arm, while novice players focused only on the ball. Similarly, Cañal-Bruland et al. (2011) indicated that expert tennis players were better than inexperienced ones in predicting the direction of the serve, using cues from the arms and the racket.

Savelsbergh et al. (2002; 2005) using eye-movement analysis techniques, examined different strategies to identify directional cues in expert and novice soccer goalkeepers. The expert players fixed their attention on the kicker’s supporting leg and foot, and thereby identified the ball’s direction more easily than its height. In starting a movement, the novice goalkeepers reacted 479 ms before the kicker’s foot made contact with the ball, contrasting with only 230 ms for elite goalkeepers. In these studies, novice players varied in their reactions with hasty movements during the anticipation phase. However, expert and novice goalkeepers did not significantly differ in reaction time.

Bideau et al. (2004) and Vignais et al. (2009) used virtual reality to study the response of elite handball goalkeepers, manipulating the amount of information that the test subject was able to gather during the ball’s throw. The results indicated that a movement’s precision diminishes when the amount of information during the anticipation phase is reduced, highlighting the relation between the information gathered during the anticipation phase and the technical execution of movements, especially the arms. Cañal-Bruland and Schmidt (2009) and Cañal-Bruland et al. (2010), also using virtual-reality technology, showed that elite goalkeepers, compared to inexperienced ones or field players, have a superior ability to determine the ball’s direction, responding appropriately, even against fakes.

In addition, the anticipatory movement by handball goalkeepers must take into account the fakes a thrower might employ. According to Fradet et al. (2004) and Van den Tillaar and Ettema (2009), the kinetic chain of handball throwers does not behave in a typical proximal-distal (P-D) sequence, since the direction of the throw can be changed at the last instant. Therefore, even if the goalkeeper can anticipate the direction of the throw, movement should ideally be delayed until it is difficult for the player to change the direction of the throw (Schorer et al., 2007; Gutiérrez-Dávila et al., 2011).

The purpose of this study was to analyze the anticipation strategies of elite team handball goalkeepers vs. inexperienced athletes. The methodology was based on data measured from the force of the goalkeeper’s reaction synchronized with high-speed cameras that analyze the goalkeeper’s anticipation in a situation where the relation between movement perception and action is preserved (Araujo and Davids, 2009). The aim of this study is to analyze certain behavioral and biomechanical differences in the movement of the player’s center of mass (CM), related to ball release, in elite vs. inexperienced goalkeepers. In this context, we hypothesize that expert goalkeepers will maintain a predetermined and efficient anticipation strategy, while inexperienced goalkeepers will show random anticipatory behavior.

## Method

### Participants

Seven elite and seven inexperienced team-handball goalkeepers and four field players were recruited for this study. The elite group was composed of team-handball goalkeepers who had played in the first division of the Spanish League (total experience = 19 ± 8 years, age = 28 ± 5 years, body height = 1.86 ± 0.03 m, body mass = 89.79 ± 9.93 kg). The inexperienced group was composed of students from the Faculty of Sport Sciences who had never participated as goalkeepers in team-handball (age = 25 ± 5 years, body height 1.80 ± 0.04 m, mass = 77.42 ± 7.29 kg). The throwers were four team-handball players, who were specialists in shooting and belonged to first division teams of the Spanish League (age = 24 ± 1 years, body height = 1.86 ± 0.05 m, mass = 86.36 ± 6.13 kg). The study was approved by the institution’s ethics committee and carried out under its ethical guidelines. All participants signed an informed consent.

### Materials and apparatus

**Figure 1:**
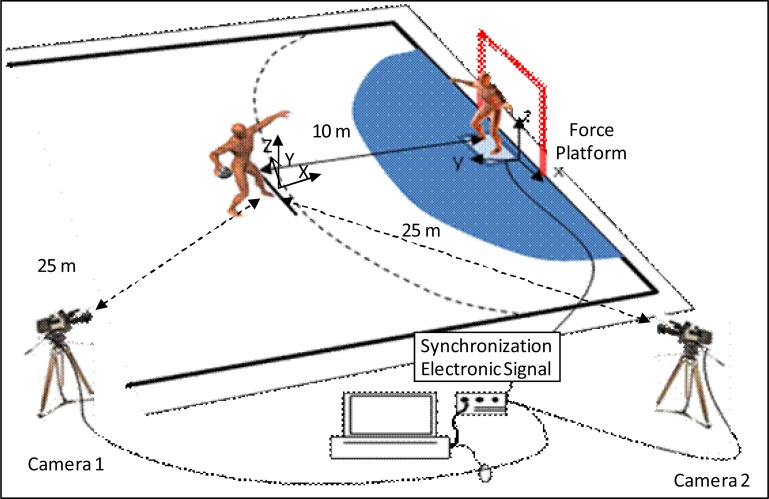
The experimental set-up used to collect data

The throws were made 10 m from the goal after a running start in a zone previously delimited by a reference system of 2.32 × 1.58 × 2 m. A force platform 0.8 × 0.8 m (Dinascan/IBV Valencia, Spain) was situated in line with the center of the goal and one meter in front of the shooting zone. The throws were filmed using two high-speed digital video cameras, Redlake MotionScope PCI 1000S (San Diego, CA, USA), at a frequency of 500 Hz, situated on the thrower’s dominant side at 25 m from the geometric center of the shooting zone and 30 m apart. This same frequency was used to record the reaction force coming from the force platform. To synchronize the two cameras and the force platform, an electronic signal was used to activate the start (Figure 1).

The three-dimensional coordinates of five body points of the thrower (point of the left foot, center of the articulations of the hip, shoulder, elbow and wrist) plus the point corresponding to the geometric center of the ball were determined for the throws chosen for analysis.

The calculations were made in three phases: a) the position of the six points (landmarks) were digitalized from the image received from the two high-speed video cameras, at a frequency of 125 Hz, b) the method of direct linear transformation was used (Abdel-Aziz and Karara, 1971) to establish the three-dimensional coordinates and c) Quintic spline functions were applied to the spatial coordinate established at this stage to smooth and interpolate the spatial coordinates at the same frequency at which they were filmed (500 Hz).

### Procedures

All goalkeepers were instructed to situate themselves in their habitual position on a force platform and not to move prior to the definitive action to save the ball. After the usual warm-up, each goalkeeper attempted to intercept 10 valid shots. The save was considered valid only when the goalkeeper moved in the correct direction to intercept the ball, marking it as an error when the goalkeeper moved to the incorrect side or stood still. After recording 10 valid actions for each goalkeeper, we analyzed the five most accurate shots (accuracy being determined by the proximity of the ball to the upper or lower corners of the goal), to determine the differences between elite and inexperienced goalkeepers.

The field players were instructed to perform 10 throws from a running start 10 m from the goal, with the sole of the front foot firmly on the ground, seeking to reach maximum velocity when releasing the ball and aiming the throw to the corners of the goal. The field players were told that they could make their usual moves before throwing, as well as changing direction during the throw if they considered it beneficial. Throws were considered valid when the player threw the ball at the goal, including the posts and the ground delimiting it.

### Dependent variables

The time of the throw (T_(THROW)_) was defined as the period between the end of the player’s run up, considered as the instant when the whole foot made full contact with the ground and the instant of that the ball leaving the player’s hand.

The velocity of the ball at release from the player’s hand (Vt_(RELEASE)_) was recorded. To determine the instantaneous tangential velocity at the moment of release, the first derivative from Quintic spline functions, with zero smoothing, was used.

The beginning of the goalkeeper’s horizontal and vertical movement related to ball release, T_START-X_ and T_START-Z_ respectively, were recorded. T_START-X_ was determined by using the data recorded from the transversal component of the reaction force, estimated at 0.001 s (half of the interval) before the instant in which net force reached a value greater than or equal to 1% of body weight. The error was determined by measuring the first 100 samples we recorded from the platform, where the goalkeeper was motionless before the player initiated the run (Gutiérrez-Dávila et al., 2006).

The instant of the start of the final movement of the vertical component of the CM (beginning of the acceleration impulse phase) related to ball release was considered to be the time when the vertical velocity of the CM became closest to zero (T_START-Z_).

The following variables were recorded: the velocity of lateral movement of the goalkeeper’s CM and the distance covered in 100 ms before the release of the ball (V_X-100_ and e_X-100_, respectively); the velocity of lateral movement, and the distance covered at the instant of ball release (V_X-REL_ and e_X-REL_, respectively); the velocity of vertical movement and the distance covered 100 ms before the ball release (V_Z-100_ and e_Z-100_, respectively); the velocity of vertical displacement and the distance covered at the instant of ball release (V_Z-REL_ and e_Z-REL_, respectively); and the maximum velocity of the vertical component during the anticipation period (V_Z-MAX_).

The instant transversal acceleration of the goalkeeper’s CM (*a*_X_) was calculated on the basis of the respective components of the goalkeeper’s net force and mass. The transversal velocity (*v*_X_) and displacement of the goalkeeper’s CM (e_X_) were calculated from the respective functions of acceleration - time, using trapezoidal integration. After normalizing the vertical component by eliminating the body weight of each goalkeeper, the same procedure was used to determine vertical velocity (*v*_z_).

### Statistics

The data were assessed for normality and homogeneity of variance, and are expressed as mean and standard deviation (SD). The mean and standard deviation (SD) of the variables were calculated for the expert and inexperienced goalkeepers. Each dependent measure was analyzed separately using a one-way analysis of variance to quantify the differences between the average scores of the expert and inexperienced goalkeepers. The analysis was performed using Statgraphics Plus 5.1. software (version 5.1). The level for acceptance of significance (α) was set at 0.05.

## Results

Table 1 sets out the descriptive statistics and the significance level between the throwers for the average tangential velocity of the ball at the instant of release from the player’s hand (*Vt*_(RELEASE)_). The results reveal differences between the players (*F*_(3,126)_= 4.44 *p* = 0.01), player 3 achieving the highest average velocity (25.43 ± 1.44 ms^−1^) and player 2 the lowest (23.88 ± 2.12 ms^−1^). In general, the mean velocity reached in all the shots analyzed (N = 129) was 24.57 ± 1.76 ms^−1^. Table 1 also shows the descriptive statistics and significance level of accuracy and the time of the shot (t_(SHOT)_) for each player. No statistically significant differences in accuracy were found among the players, while clear differences appeared in the time taken to make the shot (*F*_(3,126)_ = 6.28 *p* = 0.0005), varying between 183±16 ms for player 2 and 237 ± 23 ms for player 3, the average of all shots being 206 ± 30.3 ms.

Table 2 shows the success and mistakes expressed in percentages of the throws. The elite goalkeepers intercepted the ball in 66.3 % ± 7.5 of the throws and committed errors only in 17.5 % ± 7.6, while inexperienced goalkeepers achieved only 24.3 % ± 9.8 success with errors in 42.1 % ± 11.2 of the throws, due to factors of non-movement or movement in the wrong direction. In addition, the greater data spread for the inexperienced goalkeepers indicates more erratic behavior (Table 2)

Table 2 also shows the average, typical deviation, and statistical significance of the behavioral and biomechanical variables analyzed with elite and inexperienced goalkeepers. The data shows that elite goalkeepers’ lateral movement began −193 ± 67 ms before the ball left the hand of the thrower (T_START-X_), whereas inexperienced goalkeepers’ lateral movement began −209 ± 127 ms before. The negative value of the times indicates that the beginning of the movement occurred before the ball’s release.

The data referring to the start of the CM’s vertical shift (T_START-Z_) indicate that expert goalkeepers began this movement sooner than did inexperienced goalkeepers, the former’s average time being 77±70 ms and the latter’s 141±108 ms, both in relation to the moment of the ball’s release from the player’s hand (*F*_(1,68)_ = 7.24, *p* = 0.009).

The positive values of the data indicate that in both cases the movements began after the ball was released from the player’s hand.

No statistically significant differences were detected in the transverse component of velocity and displacement by the goalkeeper’s CM up to the moment of the ball’s release (V_X-REL_ and e_X-REL_, respectively).

The average data for the same variables 100 ms after the release of the ball (V_X-100_ and e_X-100_, respectively) were similar. Despite no significant differences between the averages, the expert goalkeepers achieved a slower transverse velocity and less displacement than did inexperienced goalkeepers. The minimal significance between the averages was due to the variability of the results for the inexperienced goalkeepers.

Table 2 also shows the data relative to the velocity and space travelled in the vertical components of the CM’s movement at the moment of the ball’s release (V_Z-REL_ and e_Z-REL_, respectively) as well as 100 ms before the release (V_Z-100_ and e_Z-100_, respectively). The measures of central tendency on the goalkeepers’ vertical movements show statistically significant differences between expert and inexperienced subjects (*F*_(1, 68)_ = 4.96, *p* = 0.03). During the anticipation period, the experts demonstrated a clear tendency to lower their CM with a slower velocity than did their counterparts (V_Z-REL_) (−0.16 ± 0.21 and −0.32 ± 0.33, respectively) and therefore moved a shorter distance at the moment of the ball’s release (e_z-REL_) (−0.03 ± 0.045m and −0.055 ± 0.085m, respectively). This lesser vertical movement of the CM in expert goalkeepers is substantiated by the values recorded for maximum vertical velocity during the anticipation phase (V_Z-MAX_), which was less for expert players than for inexperienced ones (−0.16 ± 0.22 m/s and −0.24 ± 0.42 m/s, respectively). Moreover, the spatial data as well as the data on velocity components show less dispersion in expert goalkeepers.

## Discussion and conclusions

As might be expected, the differences in the performance of both test groups confirm that the elite goalkeepers were efficient at gathering and interpreting information during the anticipation period, which was subsequently used to determine a precise intercepting movement with a higher percentage of success. However, the inexperienced goalkeepers intercepted fewer throws, found it difficult to anticipate and identify the path of the throws, and more frequently moved in incorrect directions. When they moved in correct directions, they lacked sufficient precision. These results coincide with those of Cañal-Bruland et al. (2010) and Vignais et al. (2009), who state that the ability to intercept a ball comes from precise technical execution, specifically of arm movements, and the ability to perceive cues up to the moment the ball leaves the player’s hand.

The data gathered from the start of the goalkeepers’ movements, (T_START-X_) corroborate the studies of Savelsbergh et al. (2002, 2005) in which elite goalkeepers tended to begin movement before the thrower released the ball. The minor temporal difference in elite and inexperienced goalkeepers supports the study by Vignais et al. (2009) reporting a similar response time between groups with varying experience levels. Nonetheless, the statistical values for the start of lateral movement, (T_START-X_), are lower than those of Savelsbergh et al. (2002), who measured 230 ms for soccer goalkeeper using a joystick. These differences could be attributed to the different movement structures analyzed: in our study, a complex body movement to intercept a ball, and a simple joystick movement in Savelsbergh et al. (2002).

While the average time of the throws by the four participants (T_(THROW)_) was 206 ± 30 ms, in all cases, elite as well as inexperienced goalkeeper movements commenced at the beginning of a throw (−193ms vs. −209 ms, respectively). Because of the high speeds of the ball, the goalkeepers needed to anticipate the throw in order to intercept it. The success of elite goalkeepers’ actions shows that they correctly identified the side to which the ball would be thrown based on cues from the throwers (technique and movements) before the ball was released. However, the inexperienced goalkeepers made a higher number of mistakes, implying failure to identify signals in the throwers’ technique, all of these findings agreeing with reports for other sports (Williams and Burwitz, 1993; Abernethy and Zawi, 2007).

Compared to the same data on inexperienced goalkeepers, the smaller lateral displacement of elite goalkeepers, 100 ms before the release of the ball (e_x-100_), and the subsequent slower lateral velocity of the CM (V_X-100_) indicates that elite goalkeepers may have detected certain cues indicating the direction of the throw, even though they began their movement without absolute certainty of the direction. These results coincide with the contributions of Savelsbergh et al. (2005), indicating that an elite soccer goalkeeper waits longer than an inexperienced one to decide on a reaction. This would help expert goalkeepers change their movement without signaling their reaction to the thrower.

As the movement continues, the goalkeepers subsequently increase the horizontal distance travelled (**e_X-REL_**) and the transverse velocity (V_x-REL_) until the ball’s release. At this point, it is more difficult for the player to alter the final direction of the throw without affecting its velocity (Fradet et al., 2004).

The data recorded on the vertical velocity of the goalkeepers’ CM show a clear tendency to lower the CM before the player releases the ball. The start of vertical movement (T_START-Z_) after the ball’s release timed 77 ± 70 ms for elite keepers and 141 ± 108 ms for inexperienced ones*,* which could indicate a difficulty for the goalkeeper to determine the final height of the throw. In such a case, elite goalkeepers, compared to inexperienced ones, might detect the height cues earlier. This contention is supported by the findings of Williams and Burwitz (1993) and Savelsbergh et al. (2005), who demonstrated that most errors made by soccer goalkeepers result from an incorrect perception of the factors determining shot height.

The slower movement of elite goalkeepers’ CM at the moment of the ball’s release (V_Z-REL_), the maximum vertical velocity during the anticipation period (V_Z-MAX_), and the lesser displacement of the CM (e_z-REL_), together, with the higher percentage of successful actions, indicate greater precision in the expert goalkeeper’s movements, a key factor considered by Vignais et al. (2009).

Although elite goalkeepers and inexperienced ones registered similar anticipation values for the direction of the throws (T_START-X_), most of the dispersion of the data indicates less precise and more poorly predetermined movement in the inexperienced goalkeepers. Also, their high number of errors reflects an incorrect perception of signals related to the throw direction and consequently erroneous interception movements. Meanwhile, elite goalkeepers registered a less dispersed dataset for the variables analyzed, indicating more controlled movement and more effective attention to the cues despite that throws were made in different directions.

In addition to the analysis offered here, handball goalkeeper anticipation training (like the perception training proposed by Savelsbergh et al. (2010)) could focus on detecting key movements of the throwing player, thus making it more difficult for the player to alter the direction of the ball. These results suggest that handball goalkeepers should be trained to use an anticipation strategy with the following characteristics: a) the inhibition of the primary reaction responses based on unreliable indicators; b) to start moving slowly enough to avoid being perceived by the thrower and to allow a direction change before other indicators are perceived; c) to start a precise, rapid movement only at the end of the throwing action. On the contrary, when the players throw against inexperienced goalkeepers, they should expect a quick and premature movement towards the side of the throw, based on an inadequate identification of shot direction, in addition to erratic and imprecise motor responses.

Finally, it should be indicated that anticipation strategies described are determined by the distance, speed, and type of throw used in this study. By varying these factors, the player can alter anticipation strategies and, especially, movement patterns, as suggested by Nabil and LaRue (2011).

## Figures and Tables

**Table 1:** *Descriptive Statistics and significance level of the velocity of the ball at the instant of release from the player’s hand****(Vt****_(RELEASE)_****),****and the time of shot (t_(THROW)_)*

PLAYER	1	2	3	4	*F/p*
*V*t_(RELEASE)_) (ms^−1^)	24.39 ± 1.39	23.88 ± 2.12	25.43 ± 1.44	24.71 ± 1.70	4.44**
Accuracy (m)	0.242 ± 0.117	0.225 ± 0.129	0.226 ± 0.125	0.253 ± 0.132	0.35
T_(THROW)_ (ms)	219 ± 22	183 ± 16	237 ± 23	184 ± 17	53.97***

*** p < 0.001;

** p < 0.01

**Table 2:** Descriptive analysis of balls intercepted and mistakes made by the goalkeepers as well as significant differences of biomechanical variables between elite and inexperienced goalkeepers

	Elite goalkeepers M SD	Inexperienced goalkeepers M SD	*F/P*
Intercepted Balls (%)	66.3 ± 7.5	24.3 ± 9.8	
Mistakes (%)	17.5 ± 7.6	42.1 ± 11.2	
T_START-X_ (ms)	−193 ± 67	−209 ± 127	0.38
T_START-Z_ (ms)	77 ± 70	141 ± 88	7.24**
V_X-REL_ (ms^−1^)	0.31 ± 0.20	0.32 ± 0.26	1.86
e_X-REL_ (m)	0.02 ± 0.03	0.04 ± 0.05	3.62
V_X-100_ (ms^−1^)	0.09 ± 0.12	0.15 ± 0.19	0.04
e_X-100_ (m)	0.01 ± 0.01	0.02 ± 0.03	1.18
V_Z-100_ (ms^−1^)	−0.16 ± 0.16	−0.21 ± 0.27	0.68
e_Z-100_ (m)	−0.01 ± 0.03	−0.03 ± 0.06	1.39
V_Z-REL_ (ms^−1^)	−0.16 ± 0.21	−0.32 ± 0.33	4.96*
e_Z-REL_ (m)	−0.03 ± 0.04	−0.05 ± 0.08	1.96
V_Z-MAX_ (ms^−1^)	−0.16 ± 0.22	−0.24± 0.42	0.88

** p < 0.01;

* p < 0.05
